# Pseudo-Meigs’ Syndrome in a Patient With Uterine Fibroids With Massive Pleural Effusion After Starting Gonadotropin-Releasing Hormone Agonist Therapy: A Case Report

**DOI:** 10.7759/cureus.33520

**Published:** 2023-01-08

**Authors:** Hidesato Odaka, Ruriko Asahi, Kengo Shimada, Takuo Tokairin, Yukiyo Kumazawa

**Affiliations:** 1 Department of Respiratory Medicine, Japanese Red Cross Akita Hospital, Akita, JPN; 2 Department of Pathology, Japanese Red Cross Akita Hospital, Akita, JPN; 3 Department of Obstetrics and Gynecology, Akita University Graduate School of Medicine, Akita, JPN

**Keywords:** leuprorelin acetate, massive pleural effusion, gonadotropin-releasing hormone agonist, uterine fibroids, pseudo-meigs’ syndrome

## Abstract

Pseudo-Meigs’ syndrome is caused by uterine fibroids, which is often treated using gonadotropin-releasing hormone (GnRH) agonists. Here we report a case of pseudo-Meigs’ syndrome that developed with massive pleural effusion after the initiation of GnRH agonist therapy for uterine fibroids. A 48-year-old woman presented with dyspnea. Her medical history included uterine fibroids and GnRH agonist therapy. Contrast-enhanced computed tomography revealed a massive pleural effusion, uterine fibroids, and ascites. A total laparoscopic hysterectomy was performed. The pathologic findings were consistent with those of uterine fibroids. The pleural effusion and ascites resolved completely. The patient was diagnosed with pseudo-Meigs’ syndrome due to uterine fibroids.

## Introduction

Pseudo-Meigs’ syndrome caused by uterine fibroids is rarely encountered by physicians. However, it is also included in the differential diagnosis of pleural effusion. Pseudo-Meigs’ syndrome refers to ascites and pleural fluid secondary to extra-ovarian and -broad ligament pelvic or abdominal tumors, including uterine fibroids [1]. Both ascites and hydrothorax should resolve after tumor removal [2]. On the other hand, uterine fibroids are a common gynecological disorder that can be treated using gonadotropin-releasing hormone (GnRH) agonists. Here we report a case of pseudo-Meigs’ syndrome caused by uterine fibroids after the start of GnRH agonist therapy.

## Case presentation

A 48-year-old woman with uterine fibroids received her first subcutaneous leuprorelin acetate, a GnRH agonist. The first treatment was initiated on day three of her menstrual cycle at a dose of 1.88 mg. Four weeks later, the patient received a second dose of subcutaneous leuprorelin acetate. Six weeks after the first injection, she developed dyspnea and visited her previous physician. Plain chest radiography revealed a suspected massive pleural effusion, and the patient was referred to our hospital for further examination. Her surgical history included laparoscopic myomectomy nine years prior to presentation. The patient had no history of smoking. Her father had a significant family history of colon cancer.

**Figure 1:**
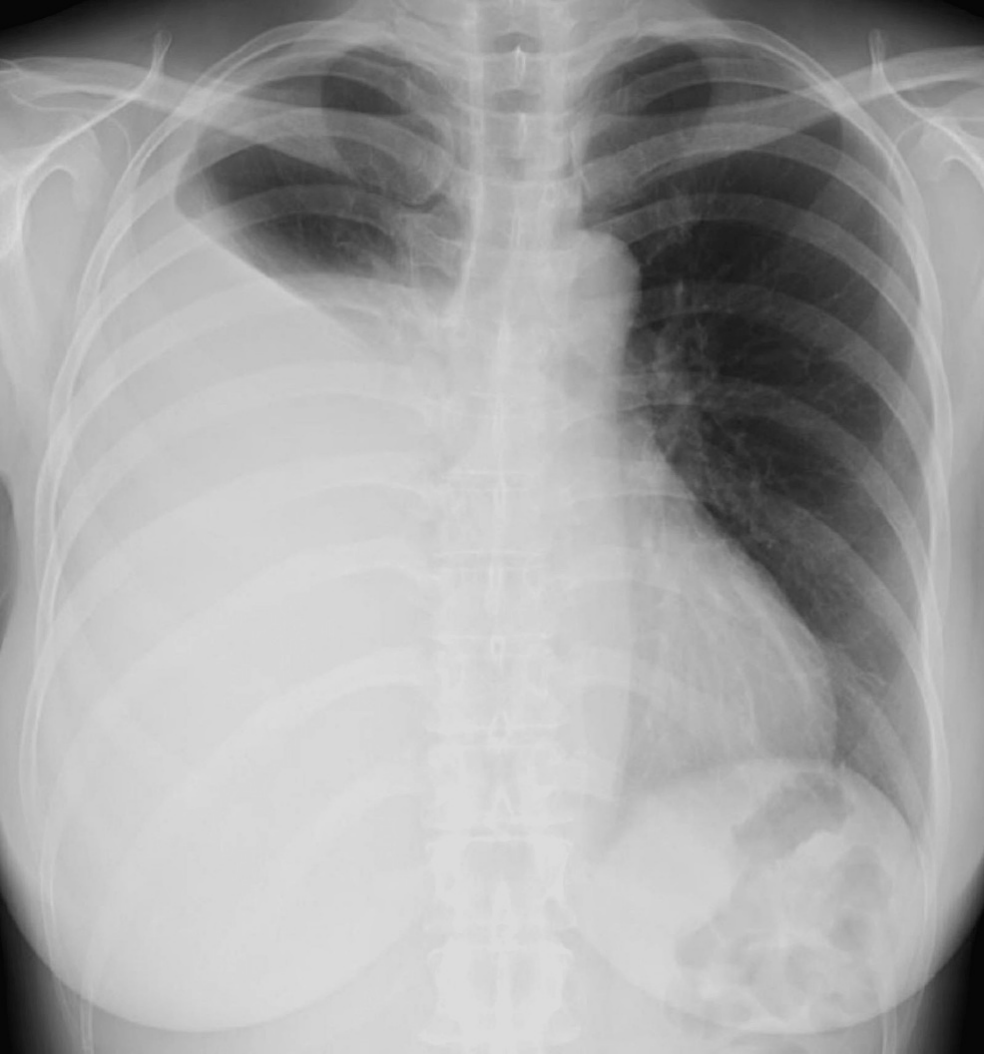
Chest X-ray obtained at the first visit revealed widespread decreased permeability of the right lung fields suggestive of a massive pleural effusion.

At the first visit, her vital parameters were as follows: blood pressure 138/87 mmHg, pulse 86 beats/min, respiratory rate 18 breaths/minute, O_2_ saturation 96% on room air, and temperature 36.7°C. No pulmonary vessel murmurs, abdominal vascular murmurs, or other remarkable physical findings were observed. Laboratory findings at the first visit (Table 1) included no inflammatory findings and no elevated lung cancer tumor markers. Almost all other findings were within the normal ranges. Plain chest radiography showed widespread decreased permeability of the right lung fields suggestive of a massive pleural effusion (Figure 1).

**Table 1:** Laboratory findings at the first hospital visit ALT: alanine transaminase; AST: aspartate aminotransferase; BUN: blood urea nitrogen; CEA: carcinoembryonic antigen; Cl: chloride; Cr: creatinine; CRP: C-reactive protein; CYFRA: cytokeratin 19 fragment; ESR: erythrocyte sedimentation rate; HCT: hematocrit; HGB: hemoglobin; K: potassium; LDH: lactate dehydrogenase; Na: sodium; PLT: platelet; Pro-GRP: pro-gastrin-releasing peptide; RBC: red blood cells; TP: total protein; T-SPOT: tuberculosis screening test; WBC: white blood cell count

Laboratory Investigation	Result	Reference Range
WBC	3,800 /μL	3,300-8,600 /μL
Neutrophils	66.3%	41.2-69.7%
Lymphocytes	23.3%	22.1-46.9%
Monocytes	6.6%	4.1-9.6%
Eosinophils	3.4%	0.0-3.5%
Basophils	0.4%	0.0-1.1%
RBC	529 × 10^4^/μL	386-492 × 10^4^/μL
HGB	15.7 g/dL	11.6-14.8 g/dL
HCT	48.0%	35.1-44.4%
PLT	27.6 × 10^4^/μL	15.8-34.8 × 10^4^/μL
ESR	2 mm/h	3-15 mm/h
TP	7.1 g/dL	6.6-8.1 g/dL
Alb	4.4 g/dL	4.1-5.1 g/dL
AST	28 IU/L	13-30 IU/L
ALT	26 IU/L	7-23 IU/L
LDH	235 IU/L	124-222 IU/L
BUN	9.6 mg/dL	8.0-20.0 mg/dL
Cr	0.73 mg/dL	0.46-0.79 mg/dL
Na	143 mEq/L	138-145 mEq/L
K	4.3 mEq/L	3.6-4.8 mEq/L
Cl	105 mEq/L	101-108 mEq/L
CRP	0.02 mg/dL	0.00-0.14 mg/dL
Anti-nuclear antibody	<40 antibody titer	< 40 antibody titer
CEA	1.2 ng/mL	≤ 5.0 ng/mL
CYFRA	1.5 ng/mL	≤ 1.5 ng/mL
Pro-GRP	50.7 pg/mL	≤ 81 pg/mL
T-SPOT	(-)	(-)

**Figure 2:**
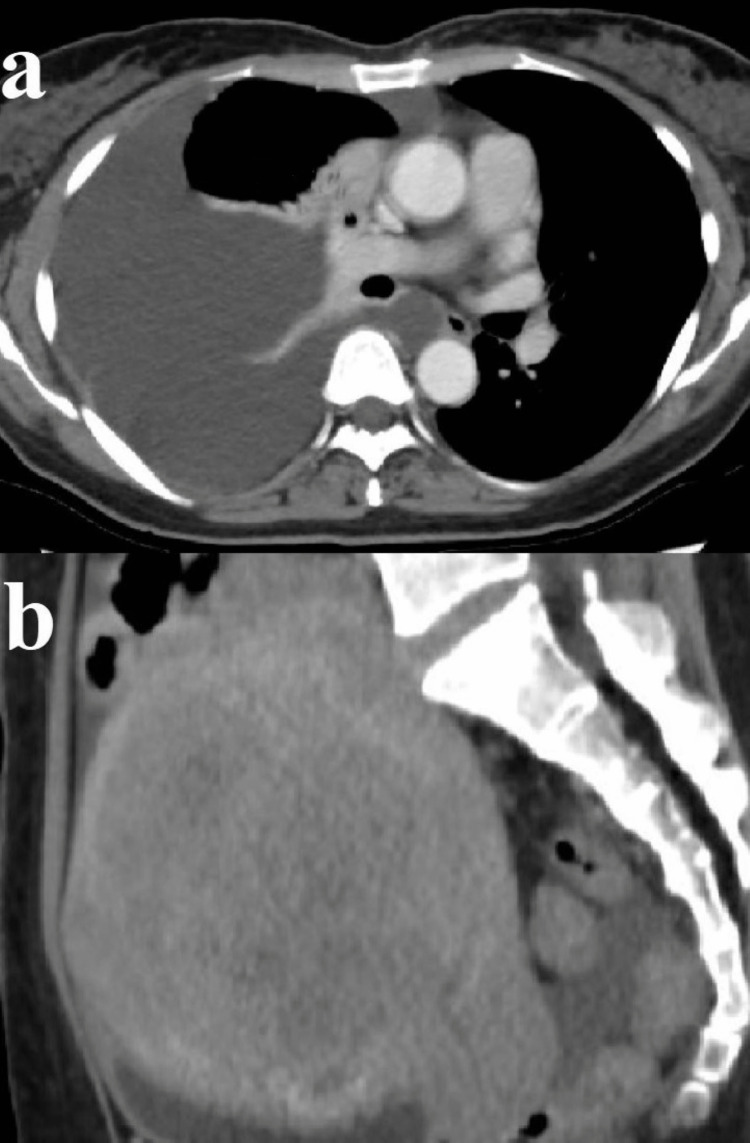
Contrast-enhanced computed tomography scan obtained during the first visit showing: (a) massive pleural effusion and (b) giant uterine fibroids and ascites in Douglas’s pouch.

Contrast-enhanced CT at the first visit showed a massive right-sided pleural effusion. Moreover, giant uterine fibroids and ascites were suspected in Douglas’s pouch (Figures 2a, 2b). 

We attempted a thoracentesis to identify the cause of the pleural effusion. The pleural effusion was exudative based on Light’s criteria (Table 2).

**Table 2:** Laboratory findings of pleural effusion ADA: adenosine deaminase; CEA: carcinoembryonic antigen; Ex: exudative; LDH: lactate dehydrogenase; T-cho: total cholesterol; Trans: transudative

Laboratory Investigation	Results	Reference Range
Colour	Bitter orange	Not available
Cell Count	104 /µL	≤ 250 /µL
Polymorphonuclear	13%	Not available
Lymphocytes	21%	Not available
Other	66%	Not available
Histiocytes	(+)	Not available
Specific gravity	1.030	Not available
Rivalta	(-)	Ex (+) Trans (-)
Protein	3.9 g/dL	Ex ≥ 3g/dL
Glucose	98 mg/dL	> 60 mg/dL
LDH	227 mg/dL	Not available
T-cho	87 mg/dL	Not available
ADA	12.6 IU/L	≤ 37 IU/L
CEA	7.3 ng/mL	Not available
Cytology	Negative	Negative
Mycobacterium tuberculosis	Negative	Negative
Bacteria	Negative	Negative

**Figure 3:**
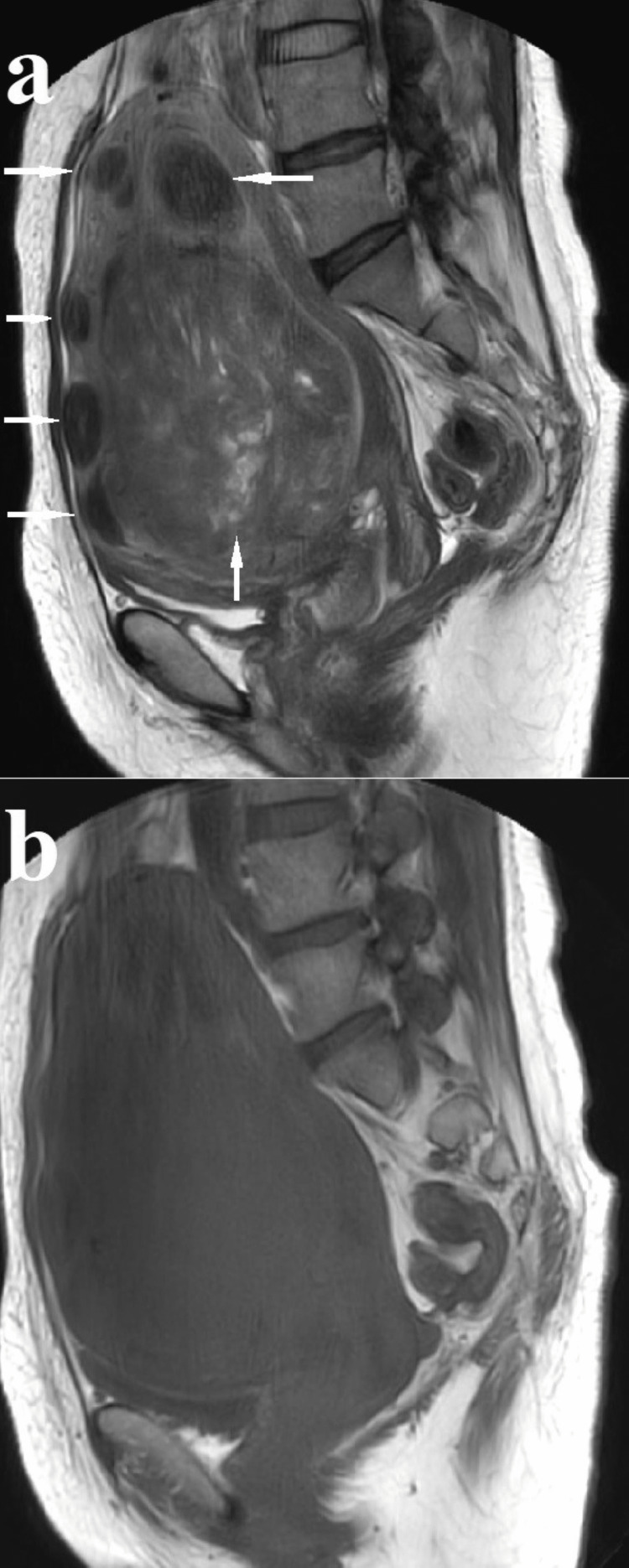
Magnetic resonance imaging of the pelvis revealed multiple uterine fibroids and no findings suggestive of malignancy (a) T2-weighted sagittal section showing 90 × 100 × 120 mm degenerative uterine fibroids submucosal to intramural in the right anterior wall of the body of the uterus (up arrow), 55 × 30 × 50 mm submucosal uterine fibroids in the fundus of the uterus (left arrow), and other multiple 10-30 mm intramural and subserous uterine fibroids (right arrow) (b) T1-weighted sagittal section showing a signal equivalent to that of the surrounding myometrium

No malignancy was observed. On magnetic resonance imaging of the pelvis, degenerative uterine fibroids were considered (Figure 3a, 3b).

**Figure 4:**
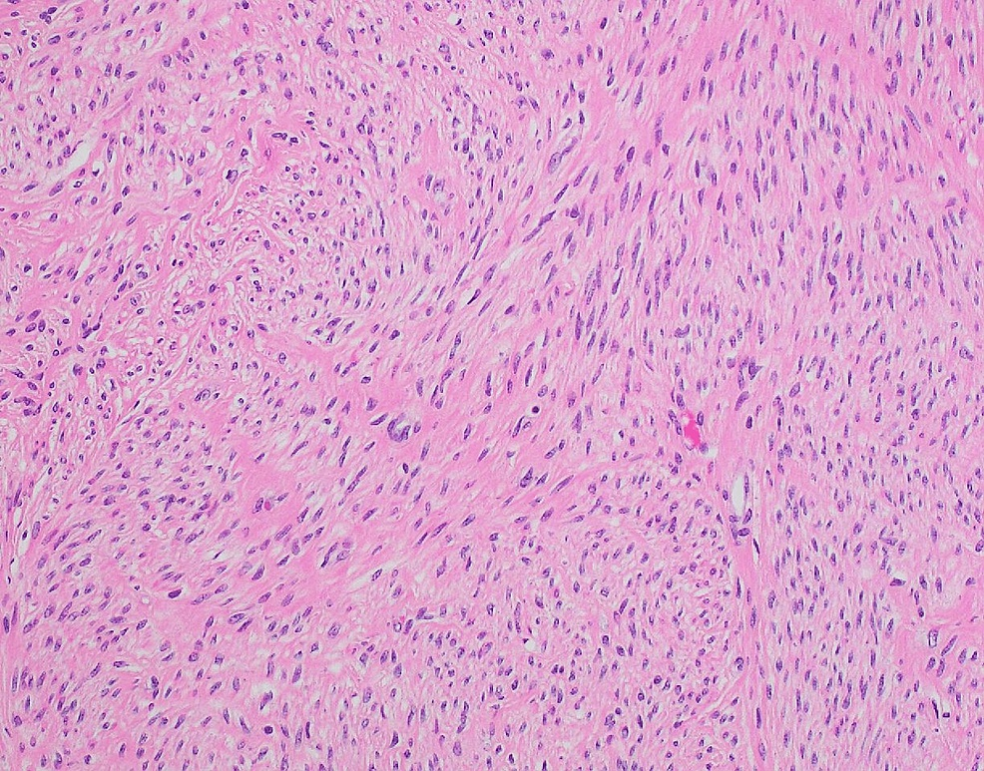
Uterine corpus (×200) showing findings of leiomyoma, that is, the bunching and complicated array of smooth muscle cells without highly atypical cells, nuclear fission, or necrosis.

Therefore, pseudo-Meigs’ syndrome was suspected for which a total laparoscopic hysterectomy was performed. The uterine weight was 700 g. A pathological examination of the uterine fibroids led to the diagnosis of smooth uterine leiomyoma (Figure 4).

The pleural effusion and ascites were completely resolved. The disease progression was consistent with pseudo-Meigs’ syndrome. Finally, the patient was diagnosed with pseudo-Meigs’ syndrome due to uterine fibroids.

## Discussion

This case report highlights two important findings. First, pseudo-Meigs’ syndrome can be caused by uterine fibroids after GnRH agonist therapy is initiated. Second, such pseudo-Meigs’ syndrome can develop with massive pleural effusion.

Some authors have postulated that the degeneration of the leiomyomata may cause the release of a substance that increases tissue permeability and results in the transudation of serous fluid from the myomas into the peritoneal cavity [3,4]. This substance is likely to be vascular endothelial growth factor [5] or interleukin-6 [6]. In our case, histologically, we could not confirm the degeneration of uterine fibroids. This may depend on how the sample was collected.

Lee et al. described the first case report of rapid development of pseudo-Meigs’ syndrome after the use of leuprolide acetate [3]. They postulated that the agonistic phase was prolonged because the patient received leuprolide acetate during the early follicular phase. In their case, the first leuprolide acetate was administered on day five of the menstrual cycle, and the onset occurred three weeks later. In contrast, in our case, the first leuprorelin acetate was administered on day three of the menstrual cycle, and onset occurred six weeks later, two weeks after the second leuprorelin acetate. The timing of the menstrual cycle of GnRH administration was similar, however, the timing of onset in our case was different from that in the other case. In our case, we were not certain that this was due to the extension of the agonist phase because we did not measure estradiol E2. The exact mechanism remains unclear; however, careful follow-up is required after the initiation of GnRH agonist therapy.

Such pseudo-Meigs’ syndrome can develop with massive pleural effusion. In the case described by Lee et al., pseudo-Meigs’ syndrome developed with massive ascites. In contrast, our patient developed massive pleural effusion. To our knowledge, no other case report has described pseudo-Meigs’ syndrome in a patient with uterine fibroids who developed massive pleural effusion after initiating GnRH agonist therapy. In our case, ascites volume was lower than pleural fluid volume. This may be due to the possibility that ascites migrated through the putative transdiaphragmatic continuity between the pleural and peritoneal spaces. Agranoff et al. reported two cases of pseudo-Meigs’ syndrome with only pleural effusion. The possible mechanism for this is the presence of a small amount of ascites, which is difficult to identify on imaging. They speculated that the ascites disappeared completely owing to continuity [7,8]. Although the details are unknown, the pleural fluid volume may be influenced by the degree of continuity.

## Conclusions

Pseudo-Meigs’ syndrome can be caused by uterine fibroids after the initiation of GnRH agonist therapy. Such pseudo-Meigs’ syndrome can develop with massive pleural effusion. We must be aware that uterine fibroids can cause pseudo-Meigs’ syndrome after the initiation of GnRH agonist therapy and pay attention to the respiratory symptoms of such patients. Future studies should determine whether uterine fibroids causing pseudo-Meigs’ syndrome after the initiation of GnRH agonist therapy are more frequent.

## References

[1] Krenke R, Maskey-Warzechowska M, Korczynski P, Zielinska-Krawczyk M, Klimiuk J, Chazan R, Light RW (2015). Pleural effusion in Meigs’ syndrome-transudate or exudate? Systematic review of the literature. Medicine (Baltimore).

[2] Saito H, Koide N, Miyagawa S (2012). Pseudo-Meigs syndrome caused by sigmoid colon cancer metastasis to the ovary. Am J Surg.

[3] Lee MJ, Kazer RR (1992). Massive ascites after leuprolide acetate administration for the treatment of leiomyomata uteri. Fertil Steril.

[4] Gal D, Buchsbaum HJ, Voet R, Forney JP (1982). Massive ascites with uterine leiomyomas and ovarian vein thrombosis. Am J Obstet Gynecol.

[5] Okuchi Y, Nagayama S, Mori Y (2010). VEGF hypersecretion as a plausible mechanism for pseudo-meigs' syndrome in advanced colorectal cancer. Jpn J Clin Oncol.

[6] Oguma T, Yamasaki N, Nakanishi K, Kinoshita D, Mitsuhashi T, Nakagawa S (2014). Pseudo-Meigs' syndrome associated with hydropic degenerating uterine leiomyoma: a case report. J Obstet Gynaecol Res.

[7] Agranoff D, May D, Jameson C, Knowles GK (1998). Pleural effusion and a pelvic mass. Postgrad Med J.

[8] Tsukao H, Ueda T, Fujii Y, Sakai T, Yamaguchi W, Nakaya J, Kojima T (2021). Incomplete pseudo-Meigs' syndrome caused by endometrial ovarian cyst: a case report. Respir Med Case Rep.

